# Urothelial carcinoma of the bladder with abundant myxoid stroma: A case report and literature review

**DOI:** 10.1097/MD.0000000000021204

**Published:** 2020-07-10

**Authors:** Ting-Ting Tao, Jun Chen, Qing Hu, Xiao-Jun Huang, Jun Fu, Bo-Dong Lv, Yue Duan

**Affiliations:** a Department of Urology, The Second Affiliated Hospital of Zhejiang Chinese Medical University, Zhejiang, China; b Zhejiang Provincial Key Laboratory of Traditional Chinese Medicine, Hangzhou, China.

**Keywords:** abundant myxoid stroma, pathology, urothelial carcinoma

## Abstract

**Introduction:**

Abundant myxoid stroma rarely occurs in urothelial carcinomas (UCs). We report an 83-year-old woman with UC of the urinary bladder with abundant myxoid stroma. We summarized the clinicopathological features, immunophenotype, diagnosis, and differential diagnosis of this type of bladder cancer, in order to improve the understanding of surgeons and pathologists.

**Patient concerns:**

An 83-year-old female presented with hematuria and frequent micturition, without odynuria, hypogastralgia, or fever.

**Diagnosis:**

The computed tomography scan demonstrated extensive tumors in the anterior wall of the bladder and a soft tissue shadow anterior to the sacrum. Cystoscopy showed massive wide-based tumors located on the anterior and lateral walls of the bladder, with no tumor involving the bladder neck. Multiple punch biopsies were performed, the histologic evaluation of which revealed a poorly differentiated invasive UCs with myxoid stroma.

**Interventions:**

The patient underwent a laparoscopic radical cystectomy and cutaneous ureterostomy.

**Outcomes:**

The patient discharged without any complications. Histologic evaluation revealed an invasive UC; the most prominent feature was an abundant myxoid stroma that covered approximately 80% of the lesion and the tumor cells were arranged in cords, small nests, or a sheet-like structure. Immunohistochemically, the tumor cells were positive for CK19, CK20, VEGF, EGFR, p63, 34βE12, MUC1, GATA3, uroplakin3, and TopII (rate = 15%), while the Ki-67 proliferation index was 10%. The myxoid stroma in the mesenchyme stained positively with AB-PAS and colloidal iron, and some tumor cells stained positive for colloidal iron. Considering the histologic, histochemical, and immunohistochemical findings, a diagnosis of UC with abundant myxoid stroma was made. After surgery, the regular follow-up was continued in clinic, and there was no recurrence for 2 years.

**Conclusion:**

Morbidity associated with UC with abundant myxoid stroma is very low. The diagnosis mainly depends on histopathological and immunohistochemical findings.

## 1 Introduction

Bladder cancer is the most common malignant tumor within the urogenital system. Bladder cancer morbidity ranks 11th globally among malignant tumors with approximately 150,000 deaths each year.^[1]^ Urothelial carcinoma (UC) accounts for > 90% of bladder cancers.^[2]^ UC of the urinary bladder has a great propensity to undergo divergent differentiation; squamous and glandular differentiation is most common,^[3,4]^ while UC with abundant myxoid stroma has been identified as a new histologic variant, as first reported by Tavora and Epstein in 2009.^[5]^ Herein, we report an 83-year-old female with UC of the urinary bladder with abundant myxoid stroma, and review the pathologic features.

## 2 Case presentation

**Figure 1:**
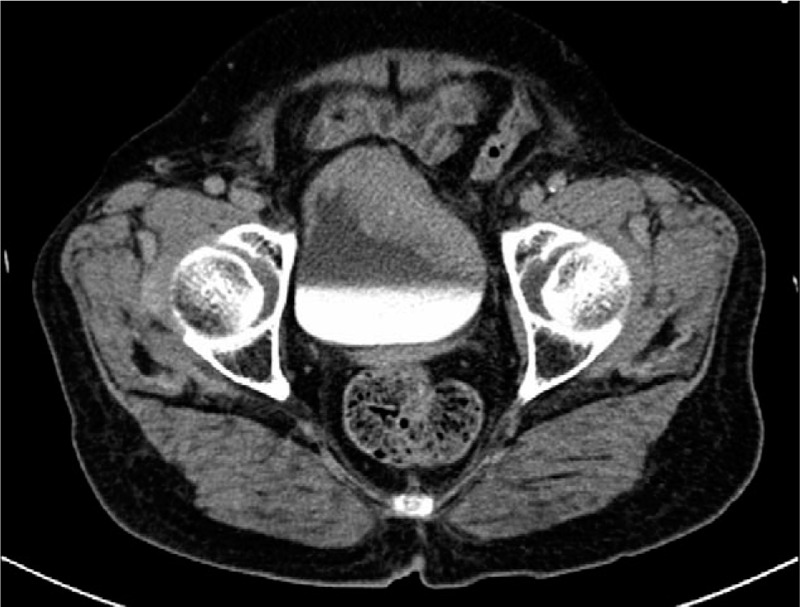
CT revealed extensive tumors in the anterior wall of the bladder.

The written informed consent was obtained from the patient for publication of this case report and accompanying images. An 83-year-old female from Hangzhou City (Zhejiang, China) was first admitted for evaluation of hematuria and frequent micturition without odynuria, hypogastralgia, or fever in May 2017. She had well-controlled hypertension for 10 years. Imaging, including CT, revealed extensive tumors in the anterior wall of the bladder and a soft tissue shadow anterior to the sacrum (Fig. 1). Cystoscopy showed massive wide-based tumors located on the anterior and lateral walls of the bladder, with no tumor involving the bladder neck. Multiple punch biopsies were performed, the histologic evaluation of which revealed a poorly differentiated invasive UC with myxoid stroma. Enteroscopy showed multiple polyps within the sigmoid colon, capillary hemangiomas in the transverse colon, and internal hemorrhoids with no evidence of metastases (clinical stage = T_3_N_0_M_0_). Considering the clinical status, a laparoscopic radical cystectomy and cutaneous ureterostomy were performed on June 6, 2017.

**Figure 2:**
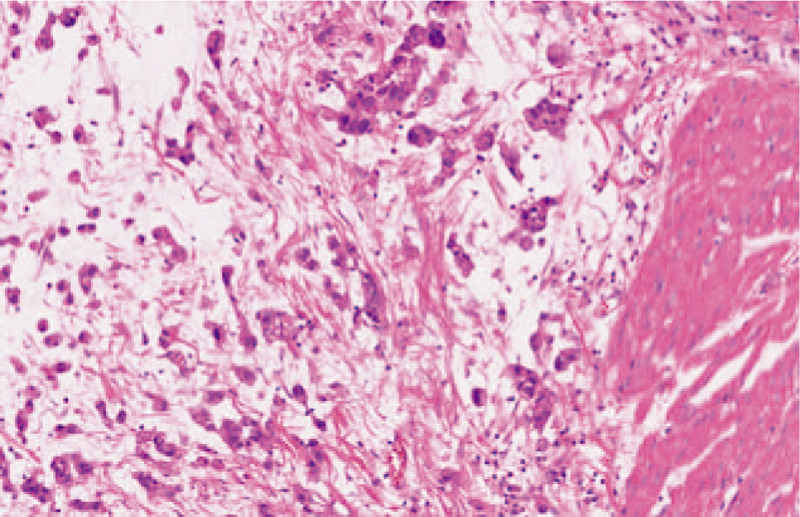
Cords and nests of tumor cells admixed with myxoid stroma, and invading smooth muscle (original magnification, ×200).

**Figure 3:**
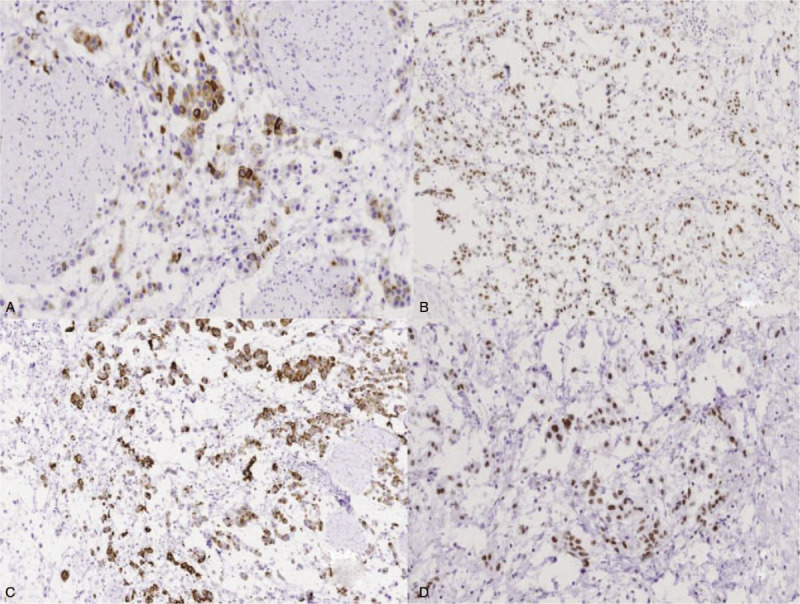
(A) The nuclei of tumor cells were GATA3-positive (original magnification, ×100). (B) The cytoplasm of tumor cells was 34βE12-positive (original magnification, ×200). (C) The cytoplasm of tumor cells was MUC1-positive (original magnification, ×200). (D) The nuclei of tumor cells were uroplakin 3-positive (original magnification, ×200).

**Figure 4:**
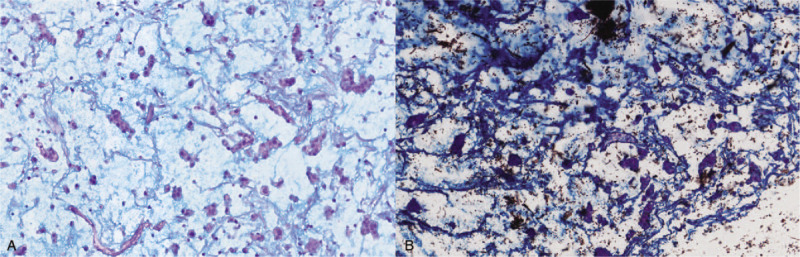
Positive staining for AB-PAS (original magnification, ×200). (A) Positive staining for colloidal iron (original magnification, ×200) (B).

The bladder was opened and nodular thickening of the bladder wall was demonstrated; the thickest area was approximately 3 cm and the cut surface was gray and gelatinous. Fresh tissue was formalin-fixed, paraffin-embedded, sectioned, stained with hematoxylin and eosin, and histochemically and immunohistochemically stained. The histologic evaluation revealed an invasive UC with abundant myxoid stroma with tumor cells infiltrating the tissues adjacent to the bladder (pT3). The tumor cells were eosinophilic with conspicuous nuclear atypia and arranged in cords, small nests, or a sheet-like structure. An abundant myxoid stroma covered approximately 80% of the lesion (Fig. 2). The tumor cells were positive for CK19, CK20, VEGF, EGFR, p63, 34βE12, MUC1, GATA3, and uroplakin3 (Fig. 3A–D). The rate of Top II expression was 15%, and the Ki67 proliferation index was 10%. Staining for CK7, MUC2, MUC5, CDX-2, villin, CD56, p53, and HER2 was negative. The myxoid stroma in the mesenchyme stained positively with AB-PAS (Fig. 4A), the background staining of colloidal iron was positive, and the cytoplasm of partial tumor cells was weakly positive (Fig. 4B).

The patient recovered without complications. Regular replacement of bilateral ureteral stents was performed. At the 2-year follow-up evaluation, CT demonstrated no recurrence.

## 3 Conclusion

UC with abundant myxoid stroma was not listed in the 2016 World Health Organization classification of tumors of the urinary tract; however, 15 cases have been reported since 2009 worldwide and the characteristics have been described in detail. The characteristic involves abundant extracellular myxoid matrix around with invasive carcinoma. Magdalena et al^[6]^ proved that urothelial cancer cells are the source of mucin, so the term “mucinous urothelial carcinoma” was thought to better characterize the lesion. The pathogenesis of UC with abundant myxoid stroma is not clear, but is thought to be related to smoking, radiotherapy, and regular exposure to chemical substances, such as benzene.

We have reviewed the clinical and pathologic data of 15 cases from the literature and the current case (11 males and 5 females) with a mean age of 69.6 ± 10.3 years (Table 1).^[5–7]^ Hematuria and dysuria were the primary symptoms. Histologically, 4 patients did not have muscularis propria involvement and the remaining patients had invasive UC. This current patient's cancer had myxoid stroma with small-sized nests and short cords. On the basis of the literature, the cancer cells in most patients were arranged in small or medium-sized nests; the others were filigree or individual cells.

**Table 1:**
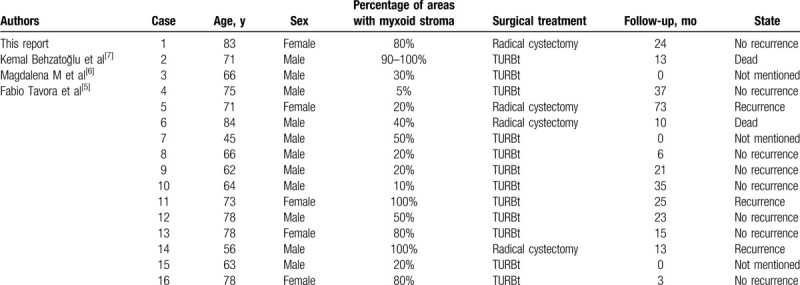
Clinical data of UC with abundant myxoid stroma.

Immunohistochemically, all cases were reactive for CK20; 15 of 16 cases were reactive for CK7 (the current patient was negative), 34βE12 was detected in 2 cases, uroplakin3 and GATA3 were detected in 1 case, and p63 was detected in 3 cases; all of the markers are relatively specific for urothelial lineage. MUC1, MUC2, and MUC5 positivity was 100% (2/2), 66.67% (2/3), and 75% (3/4), respectively. MUC1 is expressed in normal bladder mucosa, benign bladder disease, and bladder tumors; however, the expression of MUC1 was significantly increased in benign bladder lesions and bladder tumors, and the expression of bladder tumor tissue was related to the clinical staging of bladder tumor pathologic grading. Normal urinary tract epithelial cells do not express MUC2^[8]^ and MUC5,^[9]^ while MUC2 is expressed in 40% of urinary tract carcinomas. Stojnev et al^[10]^ reported that the expression of MUC2 in high-grade UC was significantly higher than low-grade noninvasive UC and was closely related to tumor prognosis. Kunze et al^[11]^ considered that the expression of *MUC5AC* gene products was significant for the transformation of UC cells into mucinous stromal adenocarcinoma. MUC2 and MUC5 were not expressed in the current patient. The rate of CDX-2 positivity was 0% (0/15). CDX-2 is a marker of gastrointestinal tumors, especially colorectal cancer, while urothelial tumors do not express CDX-2,^[12]^ which can be used to differentiate adenocarcinomas from the gastrointestinal tract. Histochemical staining with Alcian blue and colloidal iron were positive in 100% of the cases (15/15), and there was intracellular and extracellular mucus.

UC with abundant myxoid stroma should be differentiated from other diseases. First, UC with abundant myxoid stroma should be differentiated from primary mucous adenocarcinomas of the bladder and metastatic adenocarcinoma originating from the gastrointestinal tract and prostate. The latter always has glandular epithelial cells with a different degree of cytologic atypia in the mucous. Second, high-grade UC with abundant myxoid stroma should be distinguished from myxoid sarcomatoid UC, which has atypical spindle cells embedded in the mucoid stroma resembling sarcomatoid/pseudosarcomatoid stromal mucus^[13]^ and dual differentiation characteristics of both epithelium and mesenchyme.^[14]^ Third, Cox et al^[15]^ concluded that invasive UC with chordoid features was a poorly differentiated UC with myxoid stroma; most authors consider this to be a special subtype of UC.^[6,16]^ The characteristic feature was abundant myxoid stroma with tumor cells arranged in cords, which is similar to an extraskeletal myxoid chondrosarcoma. Immunohistochemically, the current case was strongly reactive for 34βE12, p63, colloidal iron, and Alcian blue without intracellular mucus. The current case was negative for CK20, AFP, and calponin. In addition, it was also necessary to differentiate the tumor from myxoid cystitis with “chordoid” lymphocytes. The morphologic features were lymphoid cells dominated by B cells arranged in cords separated by abundant mucoid matrix. Immunohistochemical analysis was negative for urothelial markers, such as CK7, CK20, p63, 34βE12, and GATA3, while lymphocyte markers, such as CD45, CD20, and CD3, were positive.

In conclusion, UC of the bladder with abundant myxoid stroma is very rare, and surgery is the main treatment. At present, the evidence of a large number of cases and long-term follow-up is limited, thus we should pay more attention to this disease in clinical practice and expect higher quality and larger data of clinical research reports to provide better evidence for the diagnosis and treatment of UC of bladder with abundant myxoid stroma.

## Acknowledgment

We wish to thank Dr De-bin Xue for help in pathologic diagnosis.

## Author contributions

**Data curation:** Qing Hu, Xiao-Jun Huang.

**Project administration:** Bo-Dong Lv, Yue Duan.

**Supervision:** Bo-Dong Lv.

**Validation:** Jun Fu.

**Writing – original draft:** Ting-Ting Tao, Jun Chen.

**Writing – review & editing:** Ting-Ting Tao, Yue Duan.
